# Mice Do Not Habituate to Metabolism Cage Housing–A Three Week Study of Male BALB/c Mice

**DOI:** 10.1371/journal.pone.0058460

**Published:** 2013-03-07

**Authors:** Otto Kalliokoski, Kirsten R. Jacobsen, Huda S. Darusman, Trine Henriksen, Allan Weimann, Henrik E. Poulsen, Jann Hau, Klas S. P. Abelson

**Affiliations:** 1 Department of Experimental Medicine, University of Copenhagen and University Hospital, Copenhagen, Denmark; 2 Department of Anatomy, Physiology and Pharmacology, Faculty of Veterinary Medicine, Bogor Agricultural University, Bogor, Indonesia; 3 Laboratory of Clinical Pharmacology, Rigshospitalet, Copenhagen, Denmark; 4 Department of Clinical Pharmacology, Bispebjerg Hospital, Copenhagen, Denmark; Oregon Health & Science University, United States of America

## Abstract

The metabolism cage is a barren, non-enriched, environment, combining a number of recognized environmental stressors. We investigated the ability of male BALB/c mice to acclimatize to this form of housing. For three weeks markers of acute and oxidative stress, as well as clinical signs of abnormality were monitored. Forced swim tests were conducted to determine whether the animals experienced behavioral despair and the serotonergic integrity was tested using an 8-OH-DPAT challenge. The metabolism cage housed mice excreted approximately tenfold higher amounts of corticosterone metabolites in feces throughout the study when compared to controls. Urinary biomarkers confirmed that these mice suffered from elevated levels of oxidative stress, and increased creatinine excretions indicated increased muscle catabolism. Changes in the core body temperature (stress-induced hyperthermia) and the fur state of the mice also indicated impaired well-being in the metabolism cage housed mice. However, monitoring body weight and feed intake was found misleading in assessing the wellbeing of mice over a longer time course, and the forced swim test was found poorly suited for studying chronic stress in mice in the present setup. In conclusion, the mice were found not to acclimatize to the metabolism cages whereby concern for animal welfare would dictate that mice should be housed in this way for as short periods as possible. The elevated degree of HPA axis activity, oxidative stress, and increased overall metabolism warrant caution when interpreting data obtained from metabolism cage housed mice, as their condition cannot be considered representative of a normal physiology.

## Introduction

Metabolism cage housing of laboratory rodents provides researchers with unique possibilities of investigating particular biological events and their progression, and it is widely used in pharmacokinetic and pharmacodynamic studies. However, it constitutes a form of single housing on wire mesh without bedding, often without enrichment, comprising of a smaller living area with no shelter. All of the aforementioned factors have been associated with induction of stress or discomfort in laboratory mice and rats, as observed in e.g. Zhu *et al.* [1] on enrichment, Ishida *et al.* [2] on the effect of living area, Bartolomucci *et al.* [3] on single housing, and Manser *et al.* [4], [5] on the effect of being housed on wire mesh. Considering that it is generally accepted that prolonged stress has profound consequences on many of the factors the metabolism cages have been designed specifically to study–e.g. the eponymous metabolism [6], [7], pharmacokinetics [8], kidney function [9], and intestinal function [10]–there are remarkably few studies on the effect of this type of housing on the wellbeing of the test subjects [11]. Whereas it has been suggested that rats may be able to adjust to metabolism cages [12]–[14], mice are considered to be less malleable and more sensitive to low-level stressors [15], [16]. Indeed some guidelines suggest that mice should be kept in metabolism cages for the shortest possible time [17]. Others are vaguer in the matter. According to the current EU directive [18], metabolism cage housing for more than 24 hours should be classed as a moderate to severe procedure with respect to pain, suffering and/or distress. On the other hand, it is common best practice to let animals habituate and acclimatize to changes in location and caging in order to obtain reliable results from subsequent experimentation [19]. In mice transferred to metabolism cages, food and water intake, urinary output, and body weight do not seem to stabilize until 3–4 days after the novel housing [20]. The stabilization of these parameters is sometimes interpreted as adjustment to the new environment. This thus provides a counterpoint, as reliable measures cannot be obtained until perhaps a week after housing mice in metabolism cages. Consequently, other guidelines recommend an acclimatization period to be included in studies in metabolism cages [21]. Unfortunately, a great majority of laboratory animal guidelines and handbooks do not address the conflict between the metabolism cages being considered stressful and the scientific need to let the animals acclimatize prior to experimentation.

The central question, which remains unanswered, is whether mice are at all capable of adjusting to the extreme environmental condition which is metabolism cage housing. We hypothesized that the mouse physiology may find a new equilibrium after a few days housing in metabolism cages, but that the stressfulness of the condition persists. This in turn means that endogenous measures obtained from laboratory mice housed in this fashion need not, and in most cases *will not*, be reflective of a normal physiology.

Acknowledging that emotional stress is a multi-dimensional construct, also in rodents [22], we used a combination of biochemical, clinical, and behavioral measures to assess the state of mice housed in metabolism cages for three weeks. We chose BALB/c mice for this study as they are the most numerous strain in our facilities, and male mice as they have been shown to better cope with single housing [23]–a condition we would obviously also impose on our control group. Traditionally measures such as body weight, feed and water consumption, defecation, and level of activity have been used to great extent to evaluate the effect of environmental stressors on laboratory mice in the past. Due to the radically different construction of the metabolism cages when compared to standard open cages, and the difference in available enrichment, activity levels are not meaningful to compare between the two cage types. Defecation is, in itself, not a useful comparative measure as the bedding material in standard cages desiccates the fecal matter greatly, reducing its mass. In the present study we opted to measure body weight, feed and water intake while also assessing fur quality, as this has been used convincingly in studies of the effect of low-intensity stressors [24].

Forcing a mouse into an inescapable adverse situation is the basis of a number of paradigms used for the induction of depressive models and a state referred to as “behavioral despair” [25], [26]. Considering that metabolism cage housing for an extended period has much in common with these models, we decided to include one of the most recognized method for assessing behavioral despair, the Forced Swim Test (FST) [27]. Forced to swim in a vessel that provides no route of escape, passive, floating, behavior exhibited by the mice may be observed. Within the paradigm, this inactivity, which is easily quantified, is interpreted as the animal giving up hope of escaping the situation; through its actions the mouse exhibits despair.

The HPA axis activity of the mice was gauged throughout the experiment by measuring immunoreactive corticosterone metabolites (CORT) excreted in feces. In addition, the integrity of the HPA axis was evaluated in conjunction with the FST. Quantifying serum concentrations of corticosterone and adrenocorticotropic hormone (ACTH) following the stressful challenge allows for detecting sensitizing/desensitizing alterations to the HPA axis both prior to, and following, adrenal integration.

A persisting stressful experience and elevated HPA axis activity are strongly correlated to elevated levels of oxidative stress [28], [29]. It is possible that even with an apparent return to normal activity of the HPA axis, free radical damage may persist. We decided to quantify oxidized metabolites of nucleic acids as these can be used as markers of intracellular oxidative stress. 8-oxo-dG (8-oxo-7,8-dihydro-2′-deoxyguanosine) is a DNA fragment excised due to mutagenic lesions brought on (purportedly) by free radical damage [30], [31]. It is readily excreted in urine together with its RNA counterpart, 8-oxo-G (8-oxo-7,8-dihydroguanosine) [32], providing two of the more accessible methods for quantifying oxidative stress [33], [34].

Chronic stress can, in addition, alter the signaling of the serotonergic system. Among other effects, a down-regulated expression of serotonin receptor 1A (5-HT_1A_) has been recognized in this context [35]. The excitability of 5-HT_1A_ in intact animals can be quantified through injections of 8-hydroxy-2-(di-n-propylamino)-tetralin (8-OH-DPAT). 8-OH-DPAT stimulates the 5-HT_1A_ receptor resulting in measurable, predictable, hypothermia proportional to the receptor density/activity [36], [37]. For simplicity we will refer to the measured induced state as HIH–hydroxy-dipropylamino tetralin induced hypothermia. Preliminary data from our laboratory indicate the method to be sensitive enough to detect the altered serotonergic signaling brought on by four weeks of single housing/social isolation in male BALB/c.

In addition to analyzing the effect of metabolism cage housing on each parameter separately, principal component analysis was employed to test for underlying trends in data. This approach circumvents the problems of multiple testing [38], while naturally incorporating the idea of stress as a multidimensional construct [22], thus being a well-suited choice for analyzing the type of data produced in the study at hand.

## Materials and Methods

### Ethics Statement

The described animal experiments were carried out on an approved license (license no. 2011/561-1980) from the Danish Animal Experiments Council, under the supervision of a local animal welfare committee at the Department of Experimental Medicine, University of Copenhagen. All efforts were made to minimize unnecessary suffering.

### Animals and Housing Conditions

Sixteen male BALB/c mice (Taconic, Ry, Denmark) of six weeks, weighing approximately 20 grams on arrival, were individually housed in Macrolon Type II cages (Tecniplast, Varese, Italy) and acclimatized to standard animal house conditions. Temperature was kept at 20 °C (Range: 18–22 °C) with 30–60% humidity and lights were set to a 12 h:12 h light cycle with lights coming on at 6∶00. Each cage was lined with Aspen chips (Tapvei Oy, Kortteinen, Finland) enriched with a cardboard hide (Brogaarden, Gentofte, Denmark), a cardboard tube (Brogaarden), wooden gnawing sticks (Tapvei Bricks; Tapvei Oy) and nest building material (Shepherd Specialty Papers, Milford, USA). Food pellets (Atromin 1319; Brogaarden) and acidified water were provided *ad libitum* throughout the entire experiment. One week after arrival, baseline HIH responsiveness was tested for. The animals remained in their home cages for the duration of the test and for a one-week washout period following the test; the washout period also allowed the animals to recover from any stress-response induced by the testing procedure. Subsequently, four randomly selected HIH low responders and four randomly selected high responders were relocated to Tecniplast mouse metabolism cages (Type 304, Stainless Steel, Cat. No. 3600M021). For the duration of the experiment all mice were housed in the same room, thus ensuring identical ambient conditions. Other animals in the room, not participating in the experiment, were housed out of sight in sound proofed air filtered units, thus ensuring that any ambient (random) stressors would relate only to the day-to-day activities in a laboratory animal facility. Metabolism cage housed mice were fed a powdered diet (Atromin 1311 FORTI; Brogaarden) with identical nutrient composition and caloric density to the control group's pellets; regular pelleted feed is incompatible with the food hoppers used in the metabolism cages. In order to best liken generic metabolism cage housing, no enrichment was provided.

No animal did at any point satisfy any of the predetermined humane endpoint conditions–a loss of body weight in excess of 20% of the starting weight, excessive dehydration, or other signs of unacceptable suffering (as judged by a veterinarian). One mouse in the control group was accidentally hurt in relation to the daily data collection on day 19, and was thus immediately removed from the study. As this did in no way affect the preceding measures obtained from the animal, data from the first 19 days have been kept for analysis.

### Daily Data and Sample Collection

Starting 8∶30 every morning all mice were weighed, as was their food and water. Feces and urine from the metabolism cages and all of the bedding in the open cages were collected and stored below −20 °C. When needed, the separation funnels and grating of the metabolism cages were changed, additional food pellets were given to the control group or water bottles were changed. Due to the low volume of urine collected in the metabolism cages, the collection tubes were carefully rinsed with 4–10 ml of deionized water to ensure that none of the sample would be lost due to evaporation. Care was taken to minimize the interactions with the animals in order for the daily routine to influence the experiment as little as possible. Due to the limited size of the feed dispensers in the metabolism cages, the powdered feed was changed daily. The feed in the dispensers was enough to last for 48 hours or possibly more, but since running low on available feed could be a potential stressor the feed was refilled more frequently.

### Fur Scoring

The first two days of the metabolism cage housing, and every three days for the remainder of the experiment, the fur quality of each mouse was assessed. The fur quality was scored on a four degree scale introduced by Mineur and collaborators [24], [39] (Table 1) by one or two observers (independently). The same two observers (veterinarians) were used throughout the experiment. Fur scoring was carried out in conjunction with the daily weighings in the vessel in which the mice were weighed. The observers were thus blinded with regards to which group the mice belonged to. We estimate that no mouse spent more than two minutes out of its home cage at any one time.

**Table 1:** Fur State Evaluation Scheme.

Score	Description
1	Well groomed fur and body. Fur is smooth and shiny with long, normal whiskers.
2	May have small areas of slightly “fluffy” patches.
3	The mouse is fluffy on most of the body. May have staining of the fur. May have abnormally trimmed whiskers.
4	The mouse is fluffy on the entire body and stained on various body parts. Bald patches and wounds may be seen. Whiskers may be trimmed. Eye conjunctivae may be red.

Definition of scores used to visually assess the state of the mice. Adopted from refs. [24], [39].

### HIH (Hydroxy-dipropylamino-tetralin Induced Hypothermia)

The density/activity of the serotonin receptor 1A (5-HT_1A_) in the mice was tested in an 8-OH-DPAT challenge (here renamed HIH, for convenience). The tested mouse was restrained and the rectal temperature was recorded with a BAT-12 temperature probe (Physitemp Instruments Inc., Clifton, USA). As soon as a stable temperature could be recorded a 2 mg/kg bodyweight subcutaneous injection of 8-OH-DPAT was given, and the mouse was returned to its home cage. The injection solution was prepared fresh less than four hours prior to the injection by sterile filtering the high activity isomer (R-(+)-8-hydroxy-2-(di-n-propylamino)-tetralin [40]) of the aminotetralin (Prod. No. H140; Sigma-Aldrich, St. Louis, USA) into a sterile isotonic saline vehicle. Care was taken to restrain the mice for as short period as possible and a stable temperature was established in less than ten seconds after insertion of the probe. Thirty minutes post-injection, when peak hypothermia through heat dissipation had been reached [37], rectal temperature was again recorded. When the HIH challenge was repeated on the last day of the experiment, it was conducted three hours after the FST (during which time all animals were single housed in Type II cages), and immediately prior to the mice being euthanized.

### Quantifying Immunoreactive Corticosterone Metabolites (CORT) in Fecal Samples

Stored frozen fecal pellets from the open cages were separated from bedding material, first through coarse sorting in an automated sieve shaker (Retsch AS 400; Retsch, Haan, Germany), and then further manually sorted. CORT was liquid extracted from pure fecal samples from metabolism cages, or from pre-sorted samples from open cages, and quantified as previously described by Sundbom *et al*. [41]. Briefly, fecal samples were submerged in 96% ethanol and incubated on a tipping table overnight. Fecal material was pelleted through centrifugation, and discarded. The supernatant was analyzed using a commercial competitive ELISA (Corticosterone ELISA, EIA-4164; DRG Instruments GmbH, Maburg, Germany) supplemented with standards (Corticosterone, Prod. No. 46148; Sigma-Aldrich) prepared in an ethanol solution. Since we feel that expressing CORT as concentrations of dry mass is misleading [42], results are presented as total CORT outputs over 24 hours.

**Figure 1:**
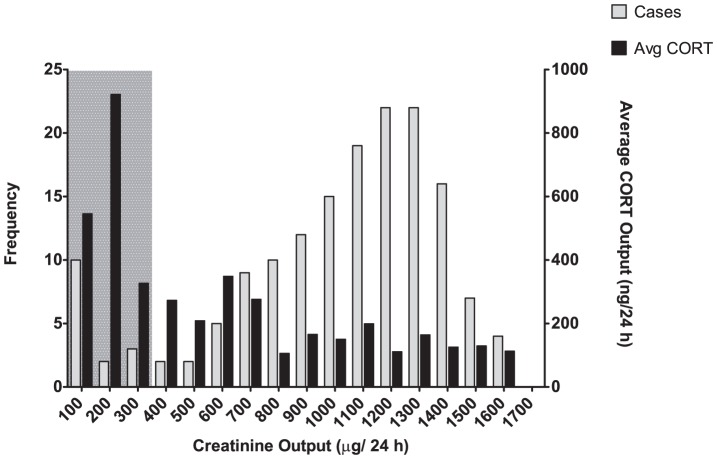
Separation failure of the metabolism cages. The number of samples (frequency of cases) and the average CORT of these samples are subdivided according to the daily creatinine excretion in the corresponding urine samples. Samples in the grey box are excluded due to the high likelihood of (severe) cross-contamination.

During the experiment, it was occasionally suspected that the separation of urine and feces in the metabolism cages had failed, allowing for cross-contamination to an unacceptable degree. Since the CORT content of urine in female BALB/c mice is known to be twice as high as that of feces [43], cross-contamination was deemed important to detect, in order to not artificially inflate the CORT output of the mice housed in metabolism cages. Data obtained where creatinine was nearly absent in urine samples, while fecal CORT was found to be significantly elevated, were flagged as questionable. A urinary creatinine cutoff was set at 300 µg/24 h (Figure 1). All samples falling below the cutoff were excluded together with the corresponding fecal samples. In all, 9.4% of all samples were excluded (26% of the total fecal CORT recovered) accounting for all the instances where, during the experiment, cross-contamination had been suspected.

### Quantifying Oxidized Nucleic Acid Metabolites and Creatinine in Urine Samples

The creatinine content in the urine samples was quantified using a colorimetric assay (ADI-907-030A; Enzo Lifesciences, Farmingdale, USA) based on the Jaffe reaction. Urine contents of oxidized nucleic acid metabolites 8-oxo-dG and 8-oxo-G were quantified using a previously described ultra-performance liquid chromatography/tandem mass spectrometry method [44]. Briefly, the chromatographic separation was performed on an Acquity UPLC system (Waters Corp., Milford, USA) using an Acquity UPLC BEH Shield RP18 column (1.7 µm, 2.1×100 mm; Waters Corp.) and a VanGuard precolumn (1,7 µm, 2,1×5 mm; Waters Corp.). The column temperature was 4 °C. The mass spectrometry detection was performed on a Xevo-TSQ triple quadrupole mass spectrometer (Waters Corp.), using electrospray ionization in the positive mode for 8-oxo-dG and negative ionization mode for 8-oxo-G.

### Forced Swim Test (FST)

On the last day of the experiment, the mice were subjected to a FST. One mouse at a time was transferred to an adjacent room which was set up for the test. The test was carried out as previously described by e.g. David *et al*. [45]. Briefly, the mouse was placed in a test cylinder (Diameter: 11 cm) filled with 23 °C water to a point where the mouse cannot sense the bottom of the cylinder (water depth: 12 cm). Observers removed themselves from the immediate vicinity of the testing cylinder to minimally influence the test (should a mouse be unable to stay afloat, experimenters still needed to be able to intervene) and video recording was started. Three video cameras were employed, set at different angles, in order to ensure that no behavior would go undetected. Six minutes after entering the testing cylinder the mice were removed, carefully dried, and placed in a pre-heated Macrolon Type II cage, placed on a heating strip (HB101; Panlab, Cornella, Spain). Video recordings were scored using the Etholog software v. 2.2 (E.B. Ottoni, Department of Experimental Psychology, University of São Paulo, Brazil) independently by two observers. The observers were thus blinded with regards to which group the mice belonged to. Two consensus scores were established for each mouse–latency to the first bout of immobility lasting more than three seconds, and total time spent immobile during the last four minutes of the test. Immobility was defined as the absence of any activity–other than movements needed to keep the head above the surface and to counter waves/creases in the water–where the mouse would remain parallel to the surface of the water. Thirty minutes after a completed FST a blood sample of approximately 200 µl was collected through submandibular venipuncture. Serum was separated and frozen until further analysis. Serum corticosterone and ACTH was determined using competitive ELISA's (EIA-4164; DRG Instruments GmbH and ACTH (Rat) EIA, EK-001-21; Phoenix Pharmaceuticals, Inc., Burlingame, USA) according to the manufacturer's instructions.

### Statistical Testing

Both serum corticosterone and CORT measures are log-normally distributed [46], wherefore they were log-transformed prior to analysis. Longitudinal parameters, e.g. body weight measures over the course of the experiment, were analyzed using repeated measures analysis of variance (ANOVA). For the measures where samples from the control group could not be obtained, analysis of covariance (ANCOVA) was employed–the day of experiment was treated as a covariate, and the individual identities were used as random explanatory variables to account for individual profiles–for testing of trends (linear increases) in excretion of creatinine and oxidized nucleic acid metabolites. The effect of time on the, supposedly, inter-related oxidative stress markers was tested in the same multivariate ANCOVA model, and their correlatedness was tested for using Pearson's product-moment correlation. Non-parametric data–e.g. FST immobility–was analyzed using the Mann-Whitney U test, and the Kruskal-Wallis rank test, when more than two groups were present. Testing of parametric data was carried out using Student's (unpaired) t-test, assuming equal variances.

In order to illustrate the problem of spot-checking the clinical signs, multiple Student's t-tests were employed for the feed and water intakes, as well as body weights. This creates the mock scenario wherein an investigator would observe the mice at a given time point during the experiment, without any knowledge of the mice's preceding states, trying in this way to assess their wellbeing.

As the underlying hypothesis acknowledged that the mice may experience different types of stress–acute, oxidative, and emotional–the inter-dependency of the measured variables was mapped through an exploratory principal component analysis. A Varimax rotation was employed in order to facilitate interpretability of the underlying components, and optimal number of components to be extracted was determined from a Scree plot.

## Results

### Body Weights, Feed and Water Intake and Fur Scores

**Figure 2:**
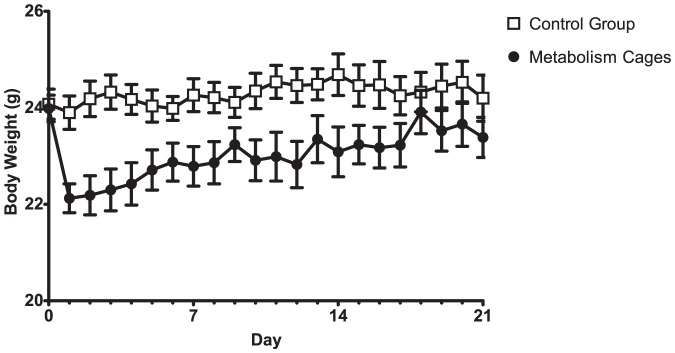
Body weights. Data are presented as mean body weights±SEM.

On the first day of the experiment the metabolism cage housed mice lost, on average, 1.9 grams (95% CI: 0.71–3.02) of body weight (approximately 8% of their starting weight) clearly separating them from the control group (T-test: t = 3.9, p<0.01). This was followed by a gradual increase (Figure 2). Body weights were indistinguishable between animals in metabolism cages and the control group from day 15 and onward (T-tests: p>0.05). An effect of the type of housing was found over time using a repeated measures ANOVA (F_1,13_ = 4.81, p<0.05).

**Figure 3:**
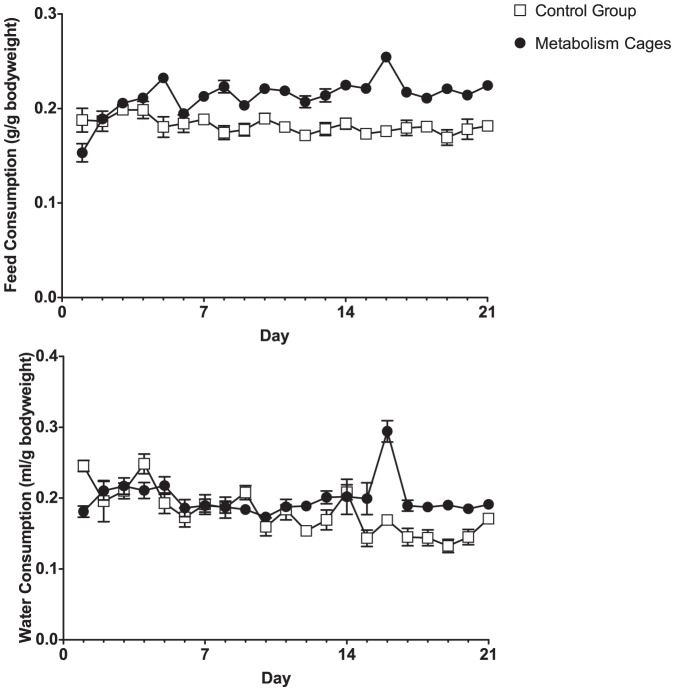
Feed and water consumption. Data are presented as means±SEM. The sudden increase in the measured parameters for day 16 is likely to be from badly calibrated scales having been used for weighing the metabolism cage feed tray and water bottles.

Feed and water intake were expressed per bodyweight to account for the differing weights of the mice following day 1. On the first day following metabolism cage housing, intake of feed and water was affected (Figure 3). Metabolism cage mice consumed significantly less (T-test: t = 2.74, p<0.05) on the first day in the novel environment. Towards the end of the experiment roles were reversed however, with the metabolism cage housed mice consuming both more feed and water than did the controls (Day 21, T-tests: Feed, t = 0.25, p<0.001; Water, t = 3.57×10^−4^, p<0.05). Consequently a change over time could be found for the metabolism cage mice, but not the control mice (repeated measures ANOVA: Housing×Day, F_1,20_ = 7.79, p<0.001).

**Figure 4:**
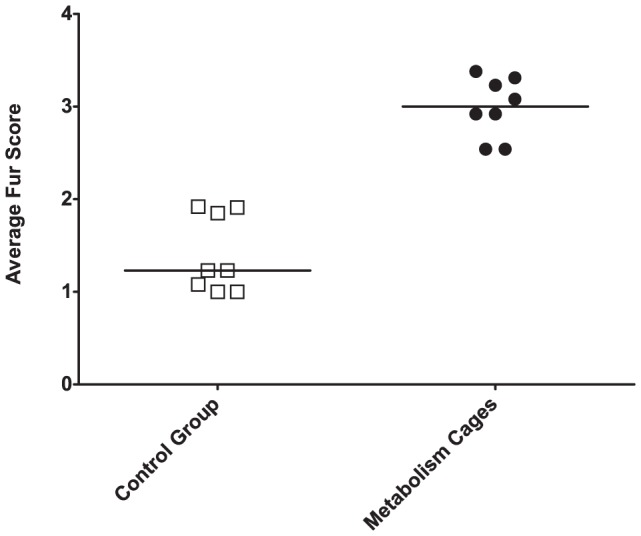
Fur scores. Average fur scores summarized from eight scoring sessions, by two independent blinded observers, spanning three weeks.

A summarized fur score was established for each mouse, every week, factoring in multiple scorings by two observers. The weekly scores were tested, group-wise, for significant changes over time (Kruskal-Wallis: χ^2^_7_ = 6.7, p = 0.46 and χ^2^_7_ = 6.3, p = 0.50 for metabolism cage mice and control, respectively). As the mice would seem to not change in the state of their fur over time, a grand score for each mouse was calculated. A clear difference between the metabolism cage housed mice and the control group could be found. A perfect separation was obtained (Figure 4) (Mann-Whitney: U = 0, p<0.001).

### Quantification of Biomarkers

All biomarker outputs were recalculated per bodyweight, as, in particular the metabolism cage housed mice, varied greatly in weight during the three weeks the study lasted.

**Figure 5:**
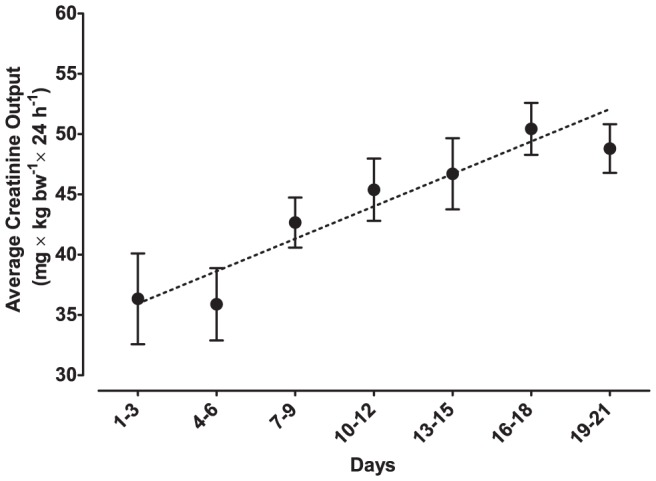
Urinary Creatinine Output of the Metabolism Cage Mice. Data are presented as means±SEM. Data are presented as averages of three days, to compensate for excluded data (refer to Results segment, and Figure 1). A significant elevation is seen over time.

**Figure 6:**
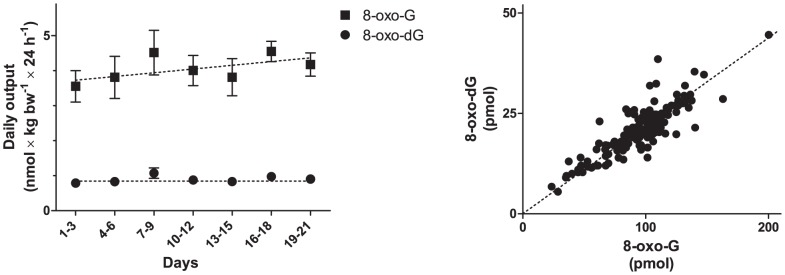
Daily urinary excretion of oxidized nucleic acid metabolites in mice in metabolism cages and correlations between the two markers. Data are given as means±SEM. Data are presented as averages of three days, to compensate for excluded data (refer to Results segment, and Figure 1). The two markers are highly correlated, but a statistically significant increase over time could only be established in the RNA-based marker (8-oxo-G).

**Figure 7:**
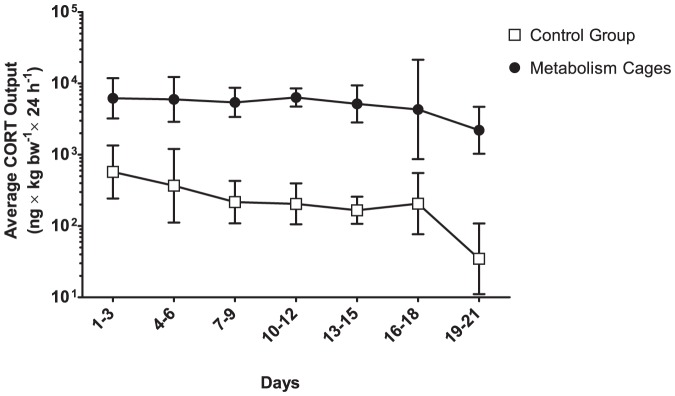
Fecal CORT output. Immunoreactive corticosterone metabolites (CORT) are quantified as nanogram-equivalents of unmetabolized corticosterone excreted within 24 hours. Data are presented as means±95% confidence intervals of the log-normal distribution. Data are presented as averages of three days, to compensate for excluded data (refer to Results segment, and Figure 1).

Mice housed in metabolism cages were found to excrete increasing amounts of creatinine towards the end of the experiment (Figure 5) (95% CI of daily increase: 0.55–1.1 mg×kg bw^−1^, F_1,145_ = 37, p<0.001). Concomitantly, this increase from day 1 was mirrored in the oxidative stress markers (F_2,143_ = 4.0, p<0.05), with a clear positive trend evident in 8-oxo-G (95% CI of daily increase: 8.3–62 pmol×kg bw^−1^, F_1,152_ = 6.7, p<0.05), but not 8-oxo-dG (F_1,152_ = 2.4, p = 0.17). Both oxidized nucleic acid metabolites were highly correlated (p<0.001; Figure 6) suggesting the increasing trend might be present in 8-oxo-dG as well, but masked by the contents of the samples approaching the detection limit. Moreover, mice housed in metabolism cages excreted approximately ten times higher amounts of fecal CORT than the control mice throughout the experiment (Figure 7) (repeated measures ANOVA following log-transformation: F_1,8_ = 1250, p<0.001).

### Forced Swim Test

**Figure 8:**
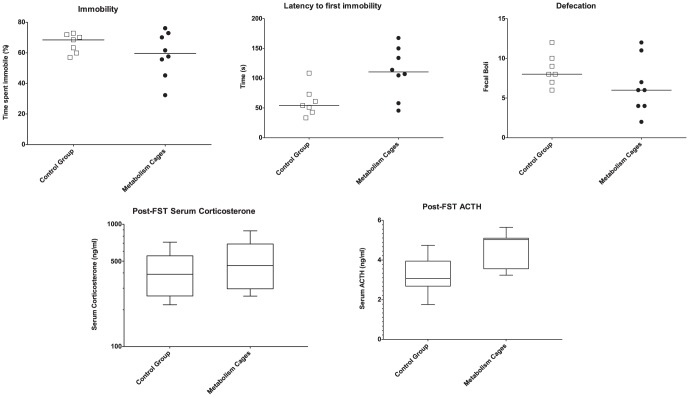
Forced swim test (FST) results summarized. Metabolism cage housed mice (n = 8) and control group (n = 7). The whiskers denote 95% CI. Significant differences were found in the latency to first immobility and the post test serum ACTH levels.

No significant difference between the mice was found in the time spent immobile during the last four minutes of the FST (Mann-Whitney: U = 21, p = 0.42). Neither was there a significant difference in serum corticosterone following the test, nor defecation during the test. Mice housed in metabolism cages were found to respond strongly in the beginning of the FST having a significantly longer latency to first immobility (Mann-Whitney: U = 10, p<0.05). They also exhibited significantly elevated levels of serum ACTH following the test (T-test: t_13_ = −2.8 p<0.05) (Figure 8).

### HIH Responsiveness

**Figure 9:**
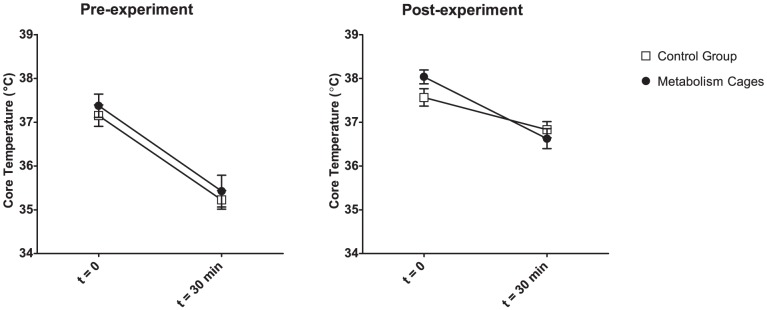
HIH (Hydroxy-dipropylamino-tetralin Induced Hypothermia). Body temperatures of the mice following an 8-OH-DPAT challenge, a week prior to the experiment and on the last day of the experiment. Data presented as means±SEM; n = 8 for all groups except the control group post-experiment, where n = 7. The pre-test temperature of the metabolism cage housed mice is significantly elevated compared to pre-experiment. The HIH post-experiment is significantly blunted for all mice.

Prior to the experiment the mice responded with an average drop in core temperature of 1.9 °C to the HIH challenge (95% CI: 0–3.8 °C). The median HIH was found to be 2.1 °C, and was used to determine high and low responders for equal distribution across the two experimental groups: Simply put, any animal with an HIH in excess of 2.1 °C was dubbed a “high responder” and any animal with an HIH less than the median was dubbed a “low responder”. No significant effect of the type of preceding housing was found in the HIH responsiveness of the mice (Figure 9), however, both groups of mice displayed a blunted response to the 8-OH-DPAT on the last day of the experiment (ANOVA with regards to HIH: Housing, F_1,30_ = 1.8 p = 0.19; Day, F_1,30_ = 8.3, p<0.01). Furthermore, the mice housed in metabolism cages had a significantly elevated core temperature, prior to the test, when compared to the control group (T-test: t_13_ = −2.3, p<0.05).

### Principal Component Analysis (PCA)

Prior to the factor reduction, a moderate level of data pre-treatment was performed. The pre-test HIH responsiveness scores had low communalities with all other variables and were thus excluded. The post-test HIH was strongly distorted by the elevated temperatures of the metabolism cage mice, and could not stand as a useful measure on its own; it was also excluded. Water intake remained at the same level throughout the experiment for both groups. Only very strongly correlated with body weights, it added nothing to the data set and was thus excluded. Maximum likelihood estimators based on the entire population were used to complete the data set. These were used *only* for the PCA, as this approach is preferable over e.g. list-wise exclusions [47].

**Figure 10:**
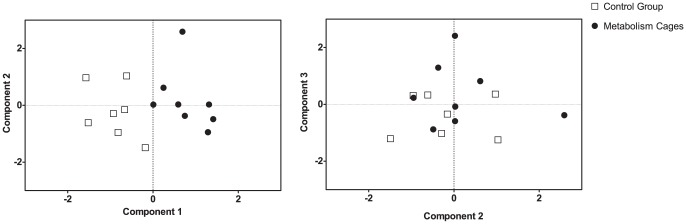
Distribution of subjects in the PCA Space. Metabolism cage housed mice (n = 8) and control group (n = 7) plotted in the 3D space defined by the extracted components. Factor loadings are presented in Table 2. A perfect separation of the experimental groups can be seen along the axis defined by component 1 (“Stress”).

Three underlying components, satisfying 75% of the variation in the tested variables, were identified (Table 2). The first component collected increased CORT output, reduced fur quality, increased feed intake, elevated body temperature, and a long latency to first immobility in the FST. There is strong precedent for these measures describing elevated HPA axis activity. The second factor illuminated a connection between increased body weight and higher levels of inactivity in the FST. The third component collected an elevated post-FST serum corticosterone and ACTH, whereas it negatively correlated with defecation during the test. This is interpreted as a coping strategy polarization in the FST. Figure 10 illustrates the distribution of the experimental populations in the derived PCA space.

**Table 2:** Factor Loadings of Extracted Components from a Principal Component Analysis (PCA).

	Component		
Initial Variables	1	2	3
Average Fur Score	0.871		
Average Feed Consumption	0.853		
Final Core Body Temperature	0.801		
Average CORT Output	0.787	0.430	
FST Latency to First Immobility	0.632		
FST Immobility		−0.800	
Final Body Weight		−0.748	
Post-FST Serum Cort			0.806
Fecal Boli During FST			−0.734
Post-FST Serum ACTH	0.451		0.671

Components, following a Varimax rotation. Only coefficients exceeding 0.4 are shown in the table below.

**Figure 11:**
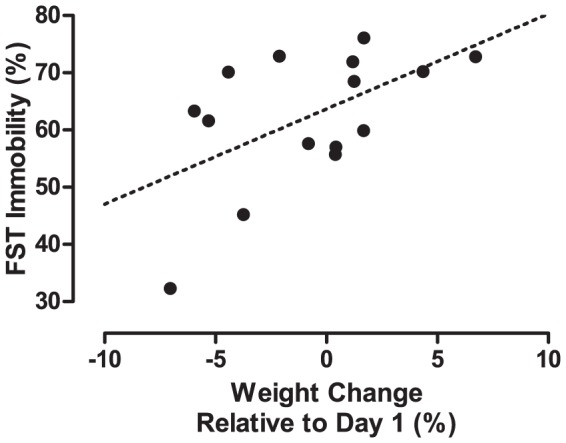
Correlation between change in body weight and immobility in the FST. Data from all 15 mice tested in the FST. A linear regression has been fitted to the data (dotted line). The variables are significantly correlated.

The discovered connection between change in body weight and immobility in the FST (Figure 11) was tested and found to be significant (Pearson's product-moment correlation: r = 0.55, p<0.05).

## Discussion

The present study illustrates the shortcomings of using feed intake, water intake, and body weight as welfare parameters on their own. Without prior knowledge, only studying the mice after day 14, an investigator would find no differences between the two groups of mice. The metabolism cage housed mice even consumed more feed, and increased somewhat faster in bodyweight, catching up with their control group counterparts. However, even when correcting data for possible cross-contaminations, the metabolism cage housed mice excreted, on average, tenfold higher amounts of CORT than did the controls. Similarly elevated HPA axis activity, persisting for as long as three weeks, is rarely encountered in literature; in our laboratory we have not previously encountered a stress response of this magnitude. The overall physiology of these animals must be considered severely affected, making them a poor model for most experimental studies.

The post-experiment elevated core body temperature provided another indication of reduced wellbeing in the mice housed in metabolism cages. Stress-induced hyperthermia has been described before–e.g. in relation to the FST in rats [25]–and has been suggested to be linked to alterations to the serotonergic system [48]. Unfortunately this could not be verified in the HIH test. As the control group displayed a similar blunted response to the test as did the metabolism cage housed mice, it is likely that the test was confounded by the preceding FST. In future studies the two tests should be mutually exclusive, or conducted further apart in time.

Thermoregulation has been shown to be important for laboratory mice [49], and depriving them of the possibility to optimize their temperature, through e.g. building nests, has been proposed to be stressful [50], [51]. The construction of the metabolism cages prevents the mice from, in any way, reducing their convective heat-loss. This has previously been proposed as the explanation to why mice maintain a higher heart rate when housed in metabolism cages [52]. An elevated degree of piloerection, as scored in the metabolism cage housed mice in the present study (Figure 4), is often interpreted as being due to less autogrooming. An alternative explanation could be that it is simply a strategy employed by the mice to minimize heat-loss to their surroundings. Regardless, evaluating the state of the animals' fur provided, in the present study, a remarkably accurate instrument for distinguishing between the control animals and those in metabolism cages. Towards the end of the experiment the body weights, and body weight gains, of the two experimental groups were undistinguishable from one-another. At the same time, mice housed in metabolism cages consumed significantly more feed than mice housed in standard cages. This is indicative of an overall higher metabolism in the former–possibly due to their having higher energy expenditure in order to maintain a normal core temperature. This elevated metabolism could in part explain why metabolism cage housed mice excreted increasing levels of creatinine and oxidized nucleic acid metabolites. The increase in creatinine excretion is evidence of a higher degree of muscle catabolism, possibly due to the overall higher metabolic rate and the severely elevated levels of circulating glucocorticoids [53]. Whereas the exact causal mechanisms of elevated free radical damage are difficult to map, the incidental evidence of their damaging potential and their correlance to elevated levels of 8-oxo-dG/8-oxo-G are well documented [54], [55]. The present study points to mice housed in metabolism cages having an unhealthy accelerated metabolism, likely to negatively impact their overall health. It can be speculated that it would be better to carry out metabolism studies of mice at temperatures matching their thermoneutral zone, or in cages designed to allow two mice to be used as the experimental unit, which would allow the mice to use behavioral thermoregulation. The potential benefit from this, however, remains to be investigated.

The results of the FST show that both groups elicited comparable levels of immobility during the last four minutes of the test. The metabolism cage housed mice showed a stronger immediate response to the test, the first bout of immobility occurring, on average, later, when compared to the control group. The metabolism cage housed mice thus acted contrarily to the behavioral despair paradigm, the preceding chronically stressful condition having an apparent potentiating effect on their acute stress response. In a similar vein, they also presented with higher serum levels of ACTH following the test. The serum corticosterone levels were comparable in the two groups and, due to the stressful nature of the test, can be expected to be near the physiological maximum [56]. The inability of circulating corticosterone to feedback-inhibit further release of ACTH, seen in the metabolism cage housed mice, has previously been described in models of chronic stress [57]. In essence, stress begets stress. This effect, termed facilitation, has previously been found to be particularly evident in elevated levels of serum ACTH and stress-induced hyperthermia [58]–both of which are observed in the present study.

The PCA served as a good tool in creating a more comprehensive picture of the experiment, and in scrutinizing the validity of the measured parameters as indicative of stress. Component 1 collected the measures that in the present setup proved indicative of physiological stress. We noted that the latency to first immobility correlated with these measures, but loaded weakly onto the component indicating that it is not a particularly good measure. Even weaker was the connection with the post-FST serum ACTH concentration. Furthermore, feed consumption sets the stressed mice apart, as they need to consume more to maintain bodyweight. This cannot be used as a parameter for evaluating stress however, as feed consumption is generally regarded as a positive activity. The stress-induced hyperthermia, fecal CORT output, and fur scorings all seem to be good tools in evaluating the wellbeing of the animals in the current setup. We suggest that these measures may prove useful in any future study geared towards improving metabolism cage housing or design. The PCA also revealed differing coping strategies. In components 2 and 3 the individual responses to the FST could be seen. The more likely a mouse was to defecate in the test, the more likely its serum corticosterone and ACTH were to be found low following the test (component 3). Similar polarizations in coping strategies have been seen in a number of behavioral tests [22], and in particular when using a PCA approach [59].

Component 2 displayed the covariance between body weight and FST immobility. Here the exploratory power of PCA became very obvious. When first stripping away the effect on body weight and FST of the stress associated parameters (Component 1), a strong latent confounder was found. The level of immobility displayed in the test correlated well to the weight lost or gained during the previous weeks (Figure 11). Full-grown at seven weeks of age, the weight changes seen in the mice during the experiment are mainly adipose tissue. This is highly incidental evidence, but we cannot help noting a certain logic in mice insulated with more adipose tissue being comfortable in adopting a passive coping style, whereas their scrawnier counterparts may opt to be more active, if only to maintain core temperature. Although outside the scope of the current study, considering the FST is primarily used to assess the effect of antidepressant agents–many of which have a hypothermic effect [60]–this is a finding worth noting. In the present setting however, the FST, it needs to be concluded, cannot be applied in assessing the wellbeing of the experimental mice. The concept of emotional despair is loosely defined and not anchored in biological processes and need not have any relation to stress.

Accounting for acute stress, oxidative stress, and clinical signs, the present study constitutes a fairly comprehensive study of the wellbeing of mice housed in metabolism cages. Although we suspect that the repeated handling of the animals may have served to further add to their stress, we know this to have contributed only to a minor degree in this study: The mice in the control group were handled to an equal extent and presented with no obvious stress responses. With respect to the usefulness of metabolism cages in yielding reliable results representative of healthy animals, the results are discouraging. A majority of the measures employed are in concordance, whereby we must conclude that metabolism cages constitute a stressful form of housing for mice, and that mice do not acclimatize to metabolism cage housing. From an animal welfare point of view, therefore, this form of housing of mice should be confined to as short periods of time as possible. Moreover, data obtained from mice housed singly in conventional barren metabolism cages should be interpreted carefully, because they have likely been obtained from significantly stressed animals with a greatly affected metabolism and physiology.
